# Arundic Acid Prevents Developmental Upregulation of S100B Expression and Inhibits Enteric Glial Development

**DOI:** 10.3389/fncel.2017.00042

**Published:** 2017-02-23

**Authors:** Marlene M. Hao, Elena Capoccia, Carla Cirillo, Werend Boesmans, Pieter Vanden Berghe

**Affiliations:** ^1^Laboratory for Enteric Neuroscience, Translational Research in GastroIntestinal Disorders, KU LeuvenLeuven, Belgium; ^2^Department of Physiology and Pharmacology, Sapienza University of RomeRome, Italy

**Keywords:** enteric nervous system, enteric glia, S100B, Sox10, enteric nervous system development, glia development

## Abstract

S100B is expressed in various types of glial cells and is involved in regulating many aspects of their function. However, little is known about its role during nervous system development. In this study, we investigated the effect of inhibiting the onset of S100B synthesis in the development of the enteric nervous system, a network of neurons and glia located in the wall of the gut that is vital for control of gastrointestinal function. Intact gut explants were taken from embryonic day (E)13.5 mice, the day before the first immunohistochemical detection of S100B, and cultured in the presence of arundic acid, an inhibitor of S100B synthesis, for 48 h. The effects on Sox10-immunoreactive enteric neural crest progenitors and Hu-immunoreactive enteric neurons were then analyzed. Culture in arundic acid reduced the proportion of Sox10+ cells and decreased cell proliferation. There was no change in the density of Hu+ enteric neurons, however, a small population of cells exhibited atypical co-expression of both Sox10 and Hu, which was not observed in control cultures. Addition of exogenous S100B to the cultures did not change Sox10+ cell numbers. Overall, our data suggest that cell-intrinsic intracellular S100B is important for maintaining Sox10 and proliferation of the developing enteric glial lineage.

## Introduction

The enteric nervous system (ENS) is a large, complex division of the autonomic nervous system. Located within the wall of the gastrointestinal tract, the ENS is vital for the control of gastrointestinal function, regulating motility, secretion, blood flow and many other activities (Furness, 2006). Enteric glial cells are integral to the function of the ENS and the gut, playing roles in various physiological and pathophysiological processes (Bush et al., 1998; Cornet et al., 2001; Cirillo et al., 2011; Gulbransen and Sharkey, 2012; Neunlist et al., 2014; Coelho-Aguiar et al., 2015; Kabouridis et al., 2015). Although several studies have examined the signaling pathways involved in glial differentiation (Nishino et al., 2010; Chalazonitis et al., 2011; Ngan et al., 2011; Mundell et al., 2012), little is currently known about what role enteric glia play during ENS development (Kabouridis et al., 2015; Le Berre-Scoul et al., 2016).

All the neurons and glia of the ENS arise from neural crest cells that migrate into the gut during development (Yntema and Hammond, 1954; Le Douarin and Teillet, 1973). These enteric neural crest-derived cells (ENCCs) mostly arise from the caudal hindbrain (“vagal”) level of the neural tube, entering the foregut at embryonic day (E)9.5 in mice and colonizing the entire length of the gut by E14.5 (Kapur et al., 1992). During migration, ENCCs undergo extensive proliferation and begin to differentiate into neurons and glia. At E10.5, a subpopulation (10–15%) of ENCCs expresses pan-neuronal markers (Baetge and Gershon, 1989; Young et al., 1999). Expression of the glial precursor marker, brain fatty acid binding protein (BFABP; Kurtz et al., 1994), can be found at E11.5 (Young et al., 2003), with expression of the glial markers S100B and glial fibrillary acidic protein (GFAP) identified at E14.5 and E16.5 respectively (Rothman et al., 1986; Young et al., 2003). Sox10, another marker of enteric glia in the mature ENS is initially found in all undifferentiated ENCCs (Young et al., 2002, 2003). It is downregulated in enteric neurons, but maintained in glial precursors and the majority of mature enteric glia (Young et al., 2003; Laranjeira et al., 2011; Boesmans et al., 2015b). During development, Sox10 expression is important for the maintenance of the neural crest progenitor pool (Paratore et al., 2001; Kim et al., 2003). Mice with homozygous mutations in *Sox10* are embryonic lethal and have a complete lack of ENCCs in their intestines, as well as other neural crest defects (Southard-Smith et al., 1998). In addition, *Sox10* expression is important for peripheral glia development (Britsch et al., 2001) and recently has been found to promote the expression of *S100B* in Schwann cell precursors to induce their differentiation (Fujiwara et al., 2014).

S100B is a member of the EF-family of Ca^2+^ binding proteins expressed in a variety of cell types, including astrocytes and enteric glia (Ferri et al., 1982; Hoff et al., 2008; Boesmans et al., 2015b). Its function has been extensively studied in astrocytes of the central nervous system (CNS) (Steiner et al., 2011; Donato et al., 2013). S100B has several intracellular binding partners (Santamaria-Kisiel et al., 2006), and in addition, is also secreted from glial cells, acting on cell surface receptors, such RAGE (receptor for advanced glycation end products) (Shashoua et al., 1984; Hofmann et al., 1999). In the CNS, expression of S100B by oligodendrocytes and astrocytes during development appears to herald the end of their proliferative capability and marks their differentiation status (Deloulme et al., 2004; Raponi et al., 2007). However, its function in ENS development has not been previously examined.

To explore the role of S100B in ENS development, we used arundic acid (ONO-2506), an agent that has been shown to inhibit S100B synthesis in astrocytes (Tateishi et al., 2002). To prevent the onset of S100B expression, explants of embryonic gut were taken from E13.5 mice, the day before the first immunohistochemical detection of S100B, and cultured in the presence of arundic acid for 48 h. Culture in arundic acid eliminated the appearance of S100B+ glial cells, and resulted in a decrease in the number of Sox10+ ENCCs, as well as reduced density and proliferation of ENCCs. There was no effect on the density of enteric neurons that express the RNA-binding protein, HuC/D (Hu), however, a small population of ENCCs exhibited co-expression of both Hu and Sox10. The mechanism of action of arundic acid has not been published, but it is known to also influence the expression of several other genes (Asano et al., 2005). However, arundic acid did not affect Sox10 immunoreactivity in adult cultures where S100B was already expressed. Our results suggest that S100B may be important for enteric glial differentiation by stimulating, or maintaining, Sox10 expression.

## Materials and Methods

### Animals

Embryonic and postnatal gut tissue were collected from *Wnt1-CRE;R26R-GCaMP3* transgenic mice, where the reporter protein GCaMP3 is expressed by all neural crest cell derivatives (Boesmans et al., 2013). *Wnt1-Cre;R26R-GCaMP3* mice were bred by mating *Wnt1-Cre* mice (RRID:MGI:2386570; Danielian et al., 1998) with *R26R-GCaMP3* mice (RRID:IMSR_JAX:014538; Zariwala et al., 2012). Midday of the day on which a plug was found was designated E0.5. Pregnant mice were killed by cervical dislocation; postnatal day (P)0 and embryonic mice were killed by decapitation. This study was carried out in accordance with European Directive 2010/63/EU and Belgian Royal Decree of 6 April 2010. The protocol was approved by the Animal Ethics Committee of the University of Leuven (project #192/2013).

### *In vitro* Organ Culture

E13.5 embryos were dissected in filter sterilized Krebs (containing the following in mM: 120.9 NaCl, 5.9 KCl, 1.2 MgCl_2_, 1.2 NaH_2_PO_4_, 14.4 NaHCO_3_, 2.5 CaCl_2_, and 11.5 glucose). The entire embryonic gut, including the stomach, small intestine and colon, was removed and pinned in small Sylgard dishes using 50 μm diameter tungsten wire, as described previously (Shyer et al., 2013). Gut explants were cultured in 2 ml of DMEM/F12 with 1% penicillin/streptomycin. Arundic acid (ONO-2506, Tocris Bioscience, catalog #4530) was dissolved in DMSO and added to the culture media. S100B (Sigma, cat #A6677) was dissolved in Krebs or Dulbecco’s phosphate buffered saline (DPBS) and also added to the culture media, either alone, or in combination with arundic acid. Appropriate vehicle control cultures were also performed. All preparations were incubated for 48 h at 37°C, 5% CO_2_ with media refreshed at 24 h.

### Adult ENS Cultures

Myenteric ENS cultures from adult mice were made as described previously (Lowette et al., 2014). Briefly, the small intestine was collected in sterile filtered Krebs, the outer longitudinal muscle with myenteric plexus were removed and digested in a mixture of collagenase type II (338 units/ml; Worthington Biochemical Corporation, CLS-2), protease (10 mg/ml; Sigma, P4693) and bovine serum albumin (4 mg/ml; Serva Electrophoresis, 11930) in Hanks’ balanced salt solution for 15 min at 37°C. The reaction was stopped by addition of Krebs with 10% fetal bovine serum (FBS), cells were washed and resuspended in DMEM/F12 with 10% FBS, 1% glutamine, 0.5% penicillin/streptomycin and plated on 18 mm coverslips. Cultures were allowed to grow for 24 h before addition of the mouse culture media, containing DMEM/F12, 1% glutamine, 0.5% penicillin/streptomycin, 0.25 μg/ml nerve growth factor (Alomone Laboratories, N-130), with 0.2% N-2 supplement (Thermofisher Scientific, 17502048) and 0.2% G-5 supplement (Thermofisher Scientific, 17503012). After 4–6 days in culture, culture medium containing either DMSO (3 μl/ml) or arundic acid (300 μM) were added for 48 h, with media changed after 24 h. Cells were then fixed for immunohistochemistry.

### Immunohistochemistry

All preparations were fixed in 4% formaldehyde (Sigma, 47629) in 0.1 M PBS and processed for immunohistochemistry as described previously (Boesmans et al., 2014). For preparations of E13.5, E14.5 and E16.5 gut, the entire gut tube was dissected for fixation. For P0, the gut was opened along the mesenteric border and pinned on Sylgard dishes before fixation. The entire mucosa and strips of circular muscle layers were then removed. All preparations were washed in PBS, blocked for 2 h at room temperature in blocking solution (PBS with 0.5% Triton X-100 and 4% donkey serum), then incubated in primary antibodies at 4°C overnight. Primary antibodies were diluted in blocking solution: rat anti-GFP (1:1000, Gentaur 04404-26); mouse anti-HuC/D (1:500, Molecular Probes A21271; RRID:AB_221448); goat anti-Sox10 (1:300, Santa Cruz Biotechnologies sc-17342; RRID:AB_2195374); rabbit anti-S100B (1:500, Dako Z0311; RRID:AB_10013383); rabbit anti-GFAP (1:500, Dako Z0334; RRID:AB_10013382); rabbit anti-BFABP (1:250, Millipore, ABN14; RRID:AB_10000325). After incubation in primary antisera, all samples were washed in PBS, then incubated in secondary antibodies for 2 h at room temperature (also diluted in blocking solution): donkey anti-rat Alexa 488 (1:1000, Molecular Probes, A21208; RRID:AB_141709); donkey anti-rabbit Alexa 488 (1:1000; Molecular Probes, A12106); donkey anti-mouse Alexa 594 (1:1000; Molecular Probes, A21203; RRID:AB_141633); donkey anti-sheep Alexa 647 (1:500, Molecular Probes, A21448; RRID:AB_1500712).

### Proliferation and Cell Death Assays

To examine proliferation, EdU (10 μM) was added to the culture media for 6 h prior to fixation. Preparations were processed for immunohistochemistry and then EdU incorporation detected using a Click-iT EdU Alexa 647 kit (Invitrogen, C10340) according to the manufacturer’s instructions. To examine cell death, a Click-iT Plus TUNEL Assay with Alexa 647 (Molecular Probes, C10619) was used according to the manufacturer’s instructions.

### Imaging and Analysis

After labeling, all preparations were imaged with either a Zeiss LSM510 or LSM780 confocal laser scanning microscope (Cell Imaging Core, KU Leuven). The ENCC network was visualized using anti-GFP antisera to recognize the GCaMP3 molecule. To examine differentiation, proliferation and cell death, single optical section images were taken (3.1 μm depth for x25 magnification or 1.0 μm for x63 magnification images) in the middle of the presumptive myenteric plexus. For embryonic gut preparations and organ cultures, one field of view from each region of the gut was imaged for analysis. For P0 gut, three fields of view were examined from the myenteric plexus at each gut region. For adult ENS cultures, two different fields of view were examined for each coverslip. The numbers of GFP+, Hu+, Sox10+ and S100B+ ENCCs were determined by visual counting using ImageJ software (NIH). All images were blinded before analysis. To examine the differences in the intensity of Sox10 immunohistochemistry, all preparations were imaged using the same parameters. The fluorescent intensity of individual Sox10+ cells was calculated using Igor Pro (Wavemetrics, Lake Oswego, OR, USA). Regions of interest (ROIs) were drawn over each cell nucleus and the average fluorescent signal was calculated for each ROI.

All population data, including graphs are presented as mean ± SEM. Unless otherwise stated, *n* refers to the number of gut preparations examined, with data collected from a minimum of two different litters of mice. Statistical analyses were performed with Prism (GraphPad software) or Microsoft Excel.

## Results

### Development of Enteric Glial Cells

**FIGURE 1:**
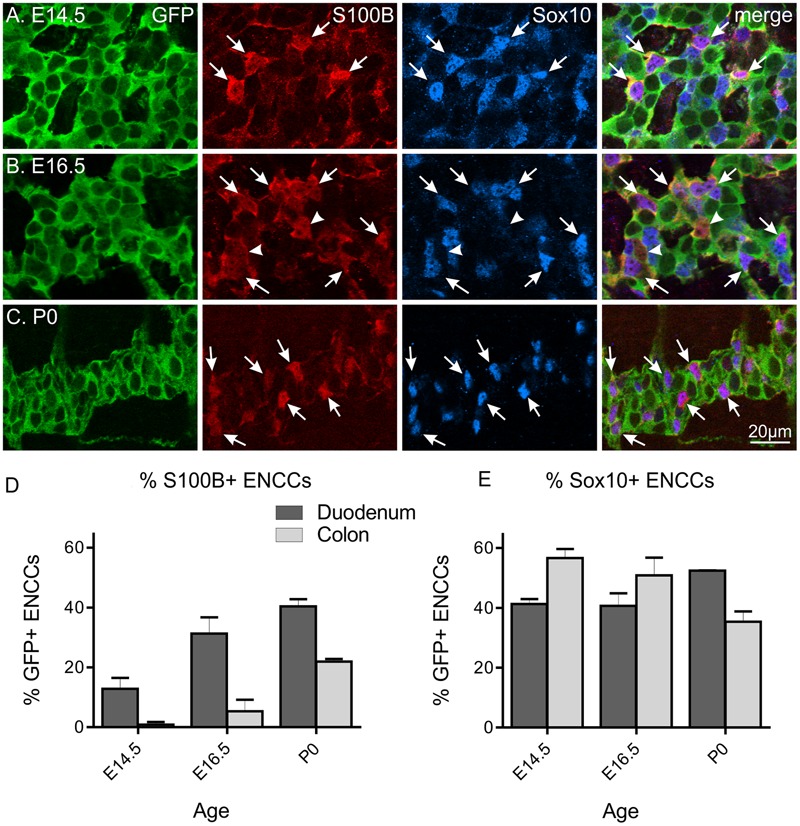
**S100B and Sox10 expression in the developing ENS of *Wnt1-CRE;R26R-GCaMP3* mice.** Representative images of the myenteric plexus in the duodenum at E14.5 **(A)**, E16.5 **(B)**, and P0 **(C)**. A small number of S100B-immunoreactive ENCCs are present in the duodenum at E14.5, which are all Sox10+ (arrows). At E16.5, a small population of S100B+ cells are not immunoreactive for Sox10 (arrowheads). Scale bar = 20 μm for all. The proportions of S100B+/GFP+ ENCCs **(D)** and Sox10+/GFP+ ENCCs **(E)** in the duodenum and colon at different ages. The proportion of S100B+ ENCCs increased during development and in general, was higher in the duodenum than the colon at each age. The proportion of Sox10+ ENCCs in the duodenum remains similar between E14.5 and P0, but decreases in the colon. Cell counts were made from *n* = 4 gut preparations for E14.5 (duodenum total of 914 GFP+ cells; colon 700), *n* = 3 for E16.5 (duodenum 503 GFP+ cells; colon 517), and *n* = 3 for P0 (duodenum 1744 GFP+ cells; colon 2614).

In order to investigate the role of enteric glia in ENS development, we first characterized the proportion of ENCCs expressing the glial marker, S100B, at various embryonic and early postnatal ages. Using *Wnt1-Cre;R26R-GCaMP3* mice, where all ENCCs express the reporter protein, GCaMP3, which can be detected using anti-GFP antisera, we examined the proportion of S100B+/GFP+ cells of the myenteric plexus in the duodenum and colon at E13.5, E14.5, E16.5 and P0 mice. At E13.5, we found no evidence of any S100B-immunoreactive cells in any region of the gut (*n* = 4, data not shown). As previously reported (Young et al., 2003), the earliest S100B-immunoreactive cells were detected at E14.5, where they made up 13 ± 4% (mean ± SEM, *n* = 4; **Figure 1**) of the total GFP+ ENCCs in the duodenum. The proportion of S100B+/GFP+ cells in the duodenum increased through development (**Figure 1**). In the colon, very few S100B+ cells were present at E14.5. A progressive increase in the proportion of S100B+/GFP+ cells was also observed in the colon from E14.5 to P0 (**Figure 1D**).

At E14.5 all S100B+ cells were also Sox10+. However, by E16.5 and P0, a small proportion of S100B+ cells were not immunoreactive for Sox10 (**Figure 1B**; 15 ± 3% for E16.5 and 6.7 ± 3% for P0 duodenum, *n* = 3 for both). In contrast, the proportion of Sox10+/GFP+ ENCCs remains fairly similar in the duodenum through late embryonic development and steadily decreases in the colon (**Figure 1E**). As Sox10 is expressed by both enteric glia as well as ENCC precursors, neuronal differentiation through development most likely contributes to this progressive decrease.

We also observed BFABP+ glial precursors at E13.5 as described previously (data not shown; Young et al., 2003), however, there was a large degree of variation in the immunohistochemical staining, and we therefore could not reliably quantify the proportion of BFABP+ ENCCs. We were also unable to obtain reliable immunolabeling of GFAP for cell counting at any embryonic age (data not shown).

### Inhibition of S100B Synthesis in Embryonic Gut Cultures Using Arundic Acid

**FIGURE 2:**
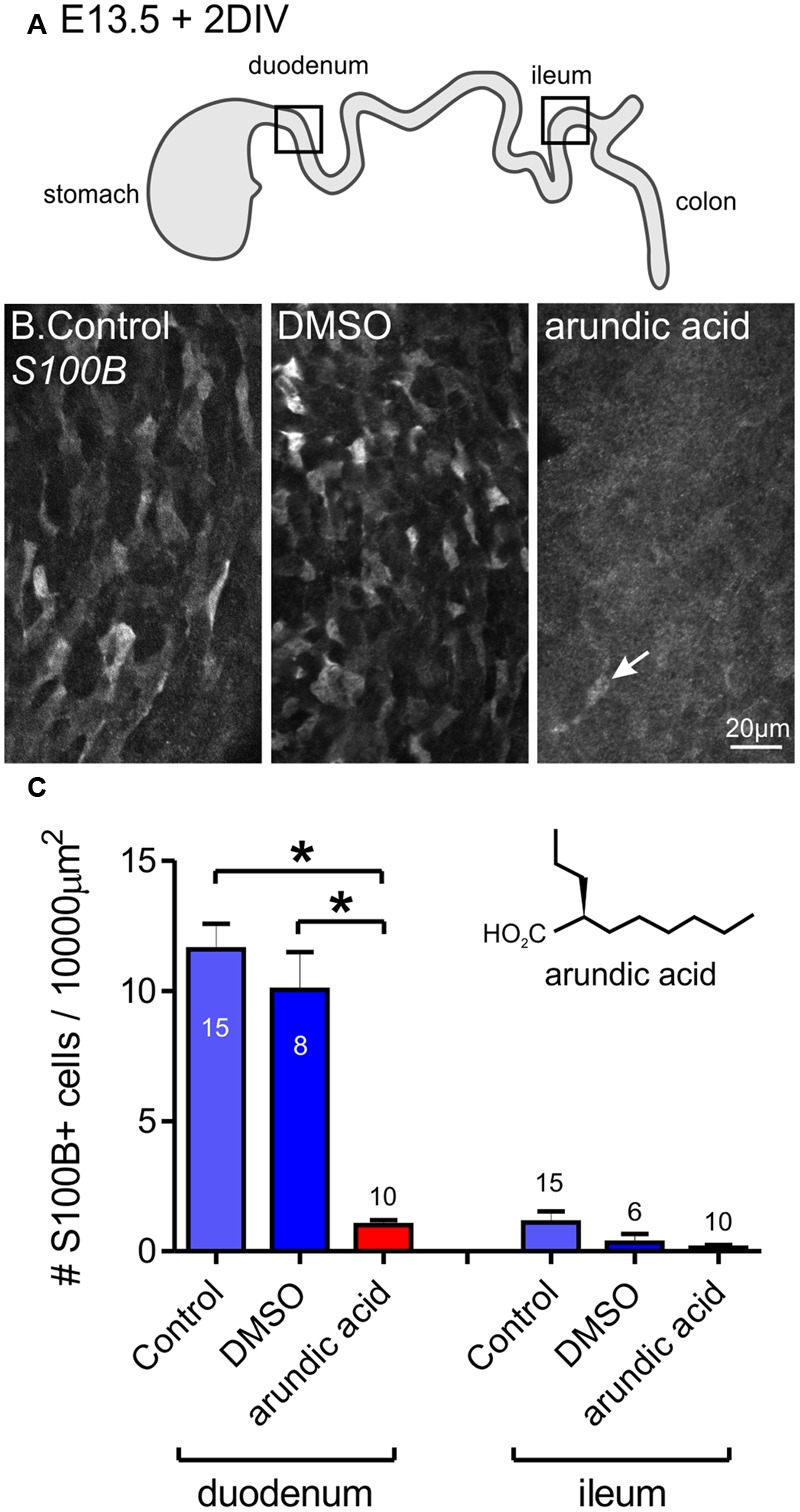
**Inhibition of S100B synthesis in the ENS using arundic acid.** E13.5 gut was cultured for 48 h in control media only, DMSO vehicle control, and arundic acid. **(A)** Diagram of the gut showing the two regions analyzed, the duodenum and ileum. **(B)** Representative images of S100B-immunoreactive cells in the duodenum after culture. Many cells were present in control and DSMO cultures. Very few S100B+ cells were present after culture in arundic acid (arrow). Scale bar = 20 μm. **(C)** The number of S100B+ cells per 10000 μm^2^ of gut area. In the duodenum, there was no obvious difference in the density of S100B+ cells in control and DMSO cultures. Almost no S100B+ cells were present in arundic acid cultures, which was significantly reduced compared to both control groups. ^∗^*p* < 0.0001, one-way ANOVA with Tukey *post hoc* test; numbers of gut preparations for each condition are shown (total S100B+ cells counted: control 571 cells; DMSO 276; arundic acid 32). In the ileum, very few S100B+ cells were observed in any of the three culture conditions (control total 49 S100B+ cells; DMSO 8; arundic acid 4). Inset shows the chemical structure of arundic acid.

To prevent S100B synthesis in the ENS, we cultured explants of embryonic gut in arundic acid, which has been shown to inhibit S100B synthesis in astrocytes (Asano et al., 2005; Cui et al., 2012; Hanada et al., 2014). E13.5 gut was used, as this was the day where no S100B-immunoreactive ENCCs were present. Because arundic acid stock was dissolved in DMSO, we also performed cultures in DMSO vehicle control (6 μl DMSO in 2 ml culture media) as well as controls with culture media only. In both control media only and DMSO vehicle control cultures, S100B-immunoreactive cells were identified in the duodenum after 48 h in culture (**Figure 2**). Arundic acid (300 μM) inhibited the appearance of S100B immunoreactivity in the ENS as no, or very few, S100B+ ENCCs could be detected after 2 days culture in its presence (**Figure 2**). In the caudal ileum, very little S100B expression was present in all three culture conditions (**Figure 2**). This is most likely due to the delay in glial differentiation along the rostral-caudal axis of the gut. A lower concentration of arundic acid (150 μM) was also examined, however, there were still abundant S100B+ ENCCs in the gut after 48 h culture in this condition (data not shown).

### The Effect of Arundic Acid on ENCC Differentiation

**FIGURE 3:**
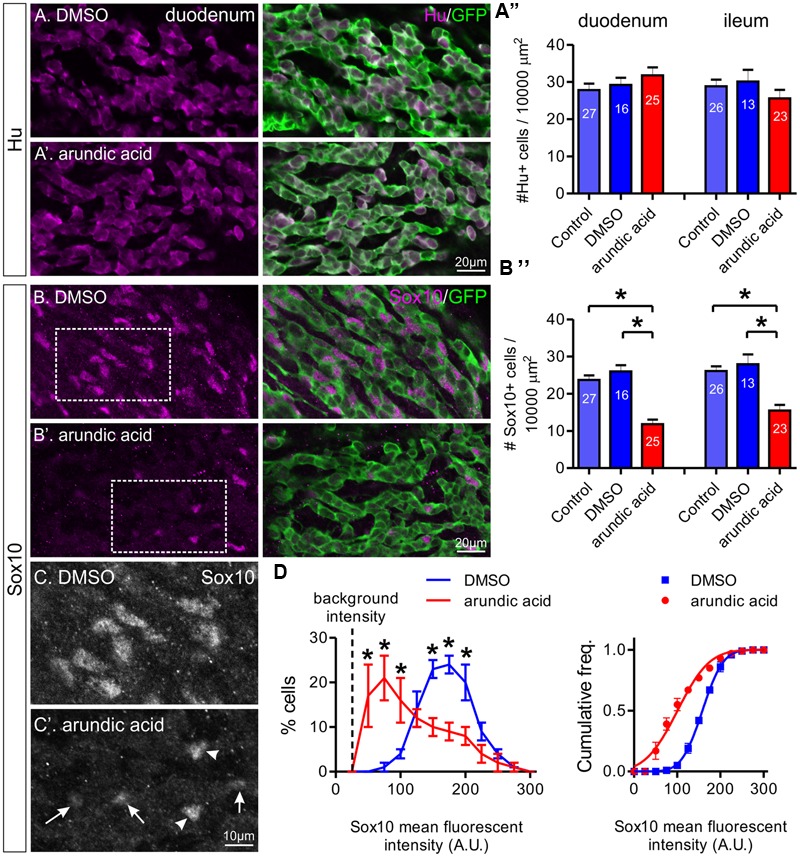
**The effect of arundic acid on Hu+ and Sox10+ ENCCs in E13.5 *Wnt1-CRE;R26R-GCaMP3* gut cultured for 48 h.** The duodenum and ileum was examined for the proportion of Hu+/GFP+ ENCCs **(A)** and Sox10+/GFP+ ENCCs **(B–D)**. Representative images of Hu+ and GFP+ ENCCs in the duodenum of DMSO **(A)** and arundic acid **(A′)** cultures. **(A″)** The density of Hu+ ENCCs did not differ for all three culture conditions in either gut region: *p* > 0.05, one-way ANOVA. *n*-numbers of gut preparations are shown (total number of Hu+ cells: duodenum control 2364; DMSO 1536; arundic acid 2743; ileum control 2249 control; DMSO 1187; arundic acid 1965). Representative images of Sox10+ and GFP+ ENCCs in the duodenum of DMSO **(B)** and arundic acid **(B′)** cultures. **(B″)** The density of Sox10+ ENCCs was significantly reduced in arundic acid cultures compared to both control and DMSO cultures in the duodenum and ileum: ^∗^*p* < 0.0001, one-way ANOVA with Tukey *post hoc* test. *n*-numbers are shown (total numbers of Sox10+ cells: duodenum control 2015; DMSO 1327; arundic acid 1010; ileum control 2050; DMSO 1090; arundic acid 1178). Scale bar = 20 μm for all. **(C,C′)** Higher magnification images of the insets from **(B,B′)** (boxes). The majority of Sox10+ ENCCs in arundic acid cultures had lower levels of fluorescent immunolabeling (arrows) compared to controls, although some cells with “normal” bright fluorescence were still present (arrowheads). Scale bar = 10 μm. **(D)** Frequency (left) and cumulative frequency (right) distributions of the fluorescent intensity profile of sox10-immunoreactive ENCCs in the duodenum of DMSO (416 cells from *n* = 5 experiments) and arundic acid (180 cells from *n* = 5 experiments) cultures. All preparations were imaged using the same parameters, the mean fluorescent intensity values of individual nuclei was calculated using Igor Pro and grouped in bins of 25 arbitrary units (A.U.). The background intensity of the gut is approximately 25 A.U. The proportion of cells in each bin from each experiment was calculated. A significantly greater proportion of cells had lower Sox10 immunofluorescence intensity (<100 A.U.) in arundic acid cultures compared to DMSO vehicle controls, whilst a lower proportion had higher fluorescent intensity immunolabeling (^∗^*p* < 0.05, two-way ANOVA with Bonferroni *post hoc* test). The cumulative frequency distribution was also different for the two populations (lines show cumulative Gaussian distribution fits).

To investigate the effect of blocking S100B synthesis on ENS development, Hu and Sox10 expression were examined in E13.5 gut explants after 2 days culture in control, DMSO and arundic acid (300 μM). Two regions of the gut were analyzed, the duodenum and ileum. After culture in control, DMSO or arundic acid, there was no obvious difference in the density of Hu+ enteric neurons in either the duodenum or ileum (**Figures 3A–A″**, one-way ANOVA, *p* > 0.05). The density of Sox10+ ENCCs was significantly decreased after culture in arundic acid in comparison to both control conditions in both gut regions (**Figures 3B–B″**, one-way ANOVA, *p* < 0.0001). In addition, in the remainder of Sox10+ cells, the intensity of Sox10 immunoreactivity appeared to be reduced in arundic acid cultures (**Figures 3B–B″,C,C′**). We therefore quantified the fluorescence intensity of Sox10 immunoreactivity in individual cells and found that the distribution profile differed for arundic acid cultures compared to DMSO vehicle controls, with a greater proportion of Sox10+ cells exhibiting lower fluorescence intensity after culture in arundic acid (**Figure 3D**, two-way ANOVA, *p* < 0.05). The cumulative frequency profile of Sox10 immunoreactivity was also significantly different for DMSO vs. arundic acid cultures, making two distinct populations with different Gaussian distribution fit parameters (**Figure 3D**, sum of squares *F*-test, *p* < 0.0001).

**FIGURE 4:**
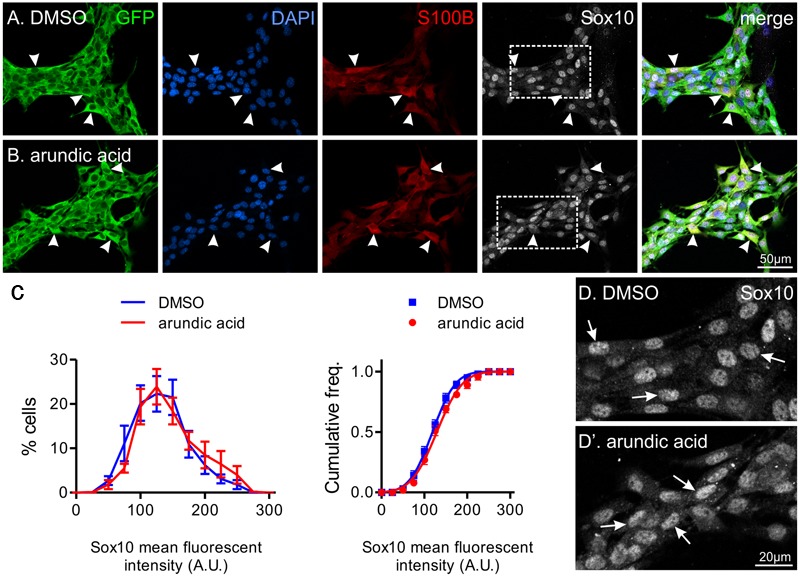
**The effect of arundic acid on Sox10 immunoreactivity in adult glial cultures.** Representative images from established mixed cultures of adult myenteric ganglia where DMSO **(A)** or arundic acid **(B)** were added for 48 h. S100B+ glia were present in both culture conditions (arrowheads) as arundic acid does not affect existing S100B protein. The majority of S100B+ glia had clear Sox10 immunoreactive nuclei (overlapping with DAPI; arrowheads). These were mixed ENS cultures and GFP+ non-glial cells were also present. Scale bar = 50 μm for all. **(C)** Frequency (left) and cumulative frequency (right) distributions of the fluorescent intensity profile of Sox10-immunoreactivity in DMSO (1459 cells from *n* = 10 coverslips from two different mice) and arundic acid (1332 cells from *n* = 10 coverslips from two different mice) cultures. The distribution of both populations is not significantly different (*p* > 0.05, two-way ANOVA). **(D,D′)** Increased magnification of Sox10-immunoreactive cells from **(A,B)** (boxes). The appearance of Sox10 immunofluorescence was similar between DMSO (D) and arundic acid **(D′)** cultures (arrows).

To ensure the changes in Sox10 immunohistochemistry was not due to a direct or S100B-independent effect of arundic acid, we also applied arundic acid (300 μM) to adult ENS cultures, where S100B+ enteric glia are already present. Arundic acid should not affect existing S100B protein, but inhibit synthesis of new protein (Asano et al., 2005). S100B-immunoreactive cells were present after 48 h culture in both DMSO and arundic acid (**Figures 4A,B**). Therefore, whilst the time-frame of S100B turnover in the adult ENS is not known, it is likely to be longer than the 48 h culture period used. There was no obvious difference in the general appearance of cells between DMSO and arundic acid cultures (**Figures 4A,B**). However, the overall density of cells was difficult to examine as GFP+ ENS cells tended to aggregate in clumps; hence, on each coverslip there where dense ENS networks interspersed with large cell-free areas. The immunofluorescence intensity of Sox10 in these S100B+ cells was not significantly different in DMSO vs. arundic acid cultures (**Figures 4C,D,D′**), indicating that arundic acid does not target Sox10 independently of S100B.

**FIGURE 5:**
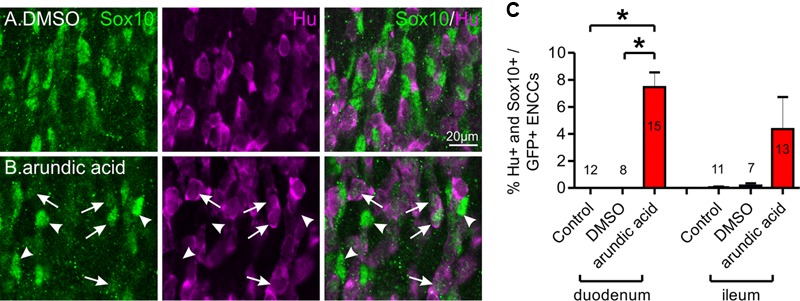
**Co-localization of Hu and Sox10 in E13.5 *Wnt1-CRE;R26R-GCaMP3* gut after 48 h culture.** Representative images of Hu+ and Sox10+ ENCCs in DMSO **(A)** and arundic acid **(B)** cultures. Very few cells were found to be immunoreactive for both Hu and Sox10 in control and DMSO vehicle control cultures. After culture in arundic acid, a subpopulation of ENCCs was found to be both Hu+ and Sox10+ (arrows), albeit the Sox10 immunoreactivity was generally not as bright in these cells. Brighter Sox10+ cells that were not immunoreactive for Hu were also present (arrowheads). Scale bar = 20 μm. **(C)** The proportion of ENCCs immunoreactive for both Hu and Sox10 was significantly greater in arundic acid cultures compared to control and DMSO cultures in the duodenum: ^∗^*p* < 0.0001, one-way ANOVA, Tukey *post hoc* test, *n*-numbers are shown. There was no significant difference in the ileum (*p* > 0.05, one-way ANOVA).

Sox10 expression is maintained in glial cells but is downregulated in neurons and generally does not co-localize with neuronal markers (Young et al., 2002, 2003; Anderson et al., 2006). However, in E13.5 gut cultured for 48 h in arundic acid, we noticed a subpopulation of ENCCs that were immunoreactive for both Hu and Sox10 (**Figure 5**). These were rarely observed in control culture conditions, but were present in both the duodenum and ileum of arundic acid cultures, although their Sox10 immunoreactivity was weaker than other Sox10+, Hu-negative ENCCs (**Figure 5**). During ENS development, a small number of Sox10+ cells with weak, cytoplasmic, but not nuclear, Hu immunoreactivity are present near the wavefront of ENCC migration as they colonize the gut (Young et al., 2004); however, the cells in our arundic acid cultures appear to be different as they have weak Sox10 immunoreactivity, and both cytoplasmic as well as nuclear, albeit weak nuclear, Hu immunoreactivity (**Figure 5** arrows).

### The Effect of Arundic Acid on ENCC Proliferation, Density, and Cell Death

**FIGURE 6:**
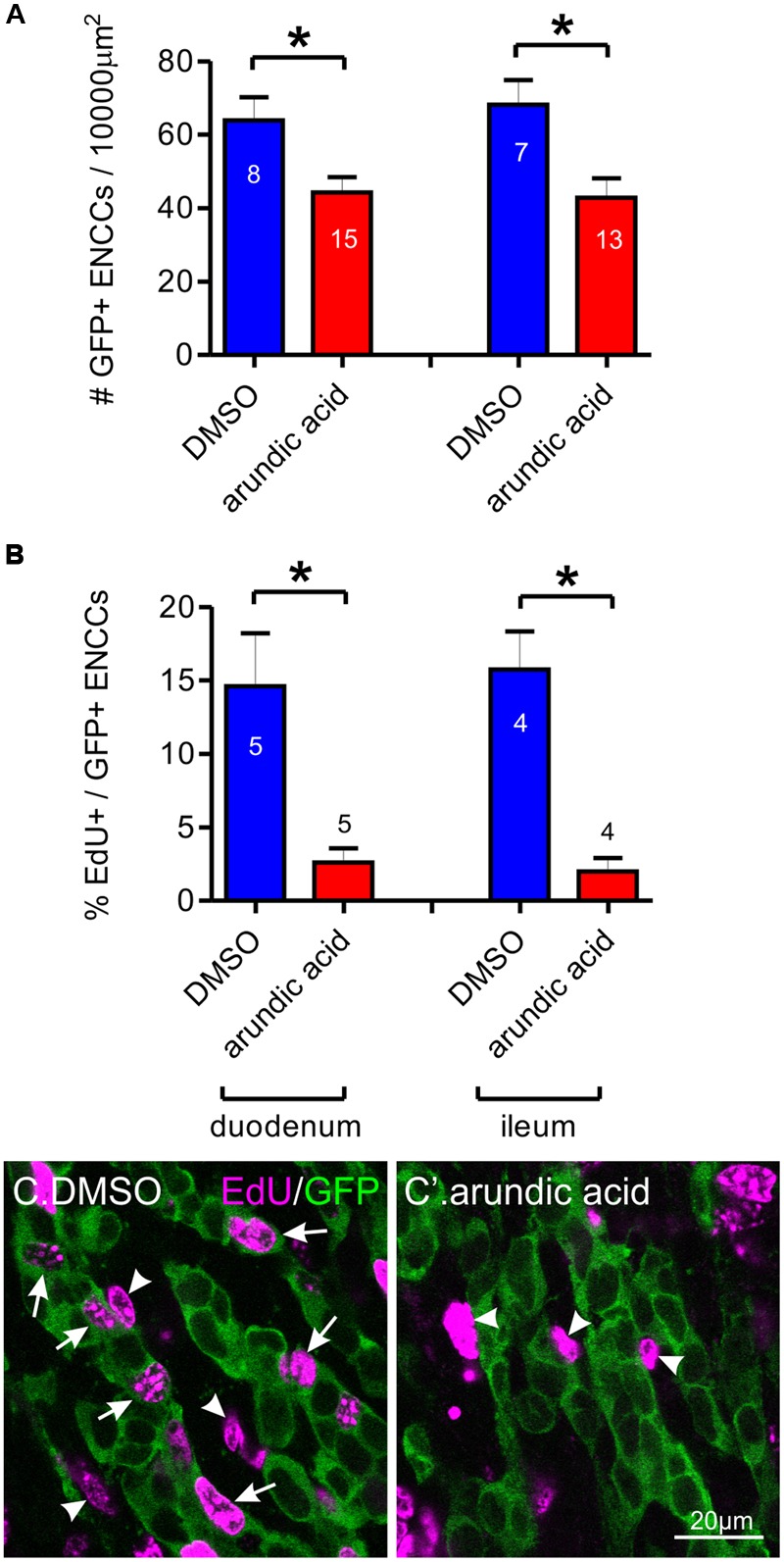
**Effect of arundic acid on ENCC density and proliferation in E13.5 *Wnt1-CRE;R26R-GCaMP3* gut after 48 h culture. (A)** The density of GFP+ ENCCs per 10000 μm^2^ was significantly reduced after culture in arundic acid compared to DMSO vehicle controls in both the duodenum and ileum: ^∗^*p* = 0.014 for duodenum, ^∗^*p* = 0.0099 for ileum, Student’s *t*-test, *n-*numbers shown on graph (total GFP+ cells counted: duodenum DMSO 1561; arundic acid 2341; ileum DMSO 1339; arundic acid 1965). **(B)** The proportion of EdU+/GFP+ ENCCS was significantly reduced in arundic acid cultures: ^∗^*p* = 0.013 for duodenum, ^∗^*p* = 0.0025 for ileum, Student’s *t*-test, *n*-numbers shown on graph. Represenative images of EdU incorporation in GFP+ ENCCs in the duodenum of DMSO **(C)** and arundic acid **(C′)** cultures. EdU+/GFP+ ENCCs were readily identifiable in DMSO cultures (arrows). There were also many EdU+ non-ENCCs that were not immunoreactive for GFP (arrowheads). In arundic acid cultures, there were fewer EdU+/GFP+ ENCCS, although EdU+/GFP-negative non-ENCCs were still present (arrowheads). Scale bar = 20 μm.

**FIGURE 7:**
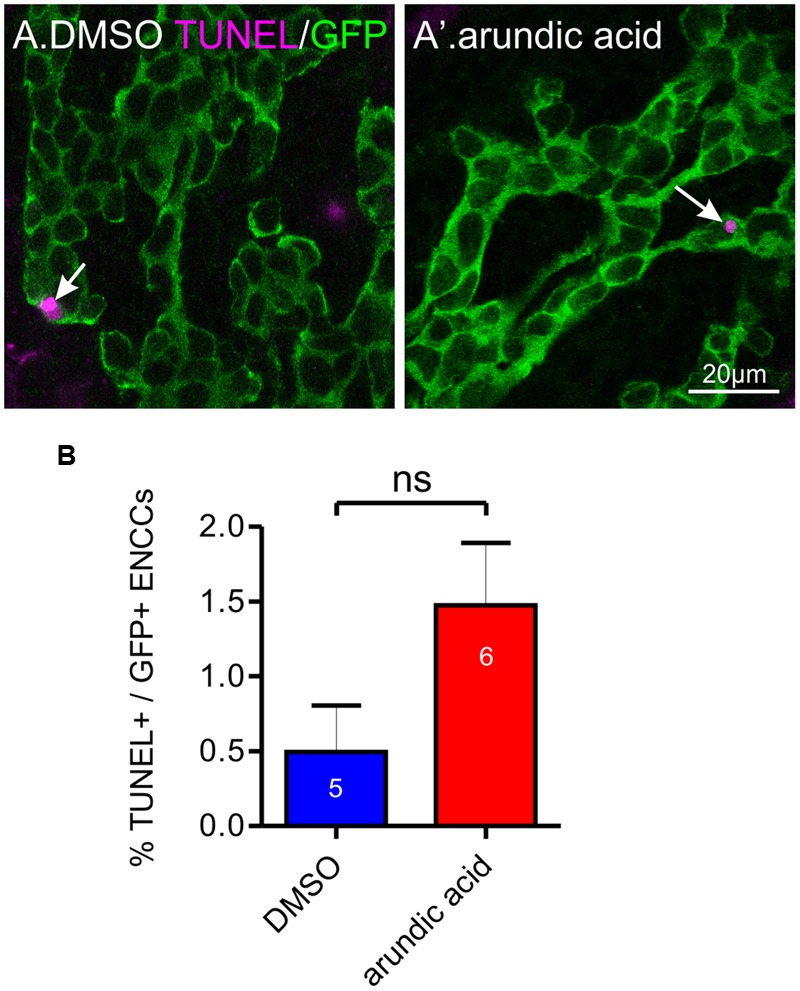
**Effect of arundic acid on ENCC cell death in E13.5 *Wnt1-CRE;R26R-GCaMP3* gut after 48 h culture.** Representative images of TUNEL staining (arrows) in the duodenum after culture in DMSO **(A)** and arundic acid **(A′)**. Scale bar = 20 μm. **(B)** The proportion of TUNEL+/GFP+ ENCCs was not statistically different in the duodenum between arundic acid and DMSO cultures (*p* > 0.05, Student’s *t*-test).

Maintenance of *Sox10* expression is important for neural crest survival and proliferation (Kapur, 1999; Kim et al., 2003). We therefore examined whether the reduced Sox10 immunoreactivity observed in our arundic acid cultures influenced the appearance of the ENCC network. The density of GFP+ ENCCs was significantly reduced in E13.5 *Wnt1-cre;R26R-GCaMP3* gut cultured in arundic acid for 48 h in comparison to vehicle controls (**Figure 6A**). We investigated whether this was due to a change in proliferation or was caused by cell death. EdU incorporation during the last 6 h of culture was used to examine cell proliferation. The proportion of EdU+/GFP+ ENCCs was significantly decreased in arundic acid cultures compared to DMSO vehicle controls (**Figures 6B,C,C′**). Co-localization of EdU with S100B was also examined in DMSO vehicle control cultures to determine whether S100B+ enteric glia are capable of continuing cell division, and 22 ± 3% of S100B+ cells were found to be also EdU+ (*n* = 5). As there were very few S100B+ cells in arundic acid cultures (**Figure 2**, total of 32 S100B+ cells from the duodenum of 10 gut preparations), we did not investigate their division in arundic acid. TUNEL staining was used to examine cell death. Only a small proportion of GFP+ ENCCs exhibited TUNEL staining in both DMSO and arundic acid cultures (**Figure 7**), which was not significantly different (*p* > 0.05, Student’s *t*-test).

### Exogenous S100B Could Not Rescue the Arundic Acid Induced Decrease in Sox10+ ENCCs

**FIGURE 8:**
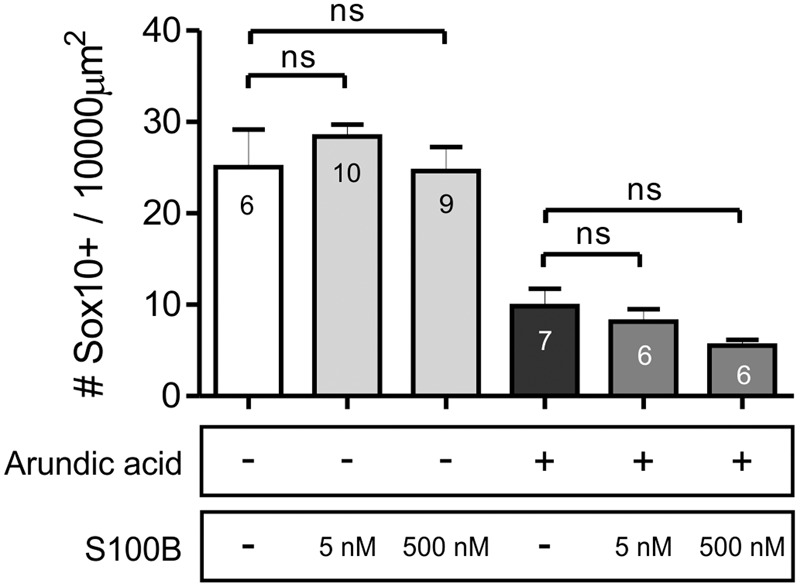
**Addition of exogenous S100B did not rescue Sox10 expression.** The density of Sox10+ ENCCs per 10000 μm^2^ of gut area was not affected by the addition of either concentration of S100B alone. Furthermore, addition of S100B with arundic acid did not change the density of Sox10+ cells in comparison to culture in arundic acid alone. *p* > 0.05 for all, one-way ANOVA, *n*-numbers shown (total number of Sox10+ cells: control 554; S100B 5 nM 702; S100B 500 nM 574; arundic acid 248; arundic acid + S100B 5 nM 266; arundic acid + S100B 500 nM 187).

To examine whether the change in Sox10+ ENCCs was mediated by cell-intrinsic or paracrine actions of S100B, we investigated the effect of adding exogenous S100B to our E13.5 gut explant cultures. Two different concentrations of S100B were used, 5 and 500 nM (Cirillo et al., 2011), and added either in combination with arundic acid, or alone. Therefore, E13.5 *Wnt1-CRE;R26R-GCaMP3* gut was cultured for 48 h in six different culture conditions: in vehicle control, S100B (5 nM), S100B (500 nM), arundic acid (300 μM) only, arundic acid + S100B (5 nM), and arundic acid + S100B (500 nM). Addition of either concentration of S100B alone did not affect Sox10+ ENCCs compared to vehicle control (**Figure 8**). Addition of S100B with arundic acid also did not change Sox10+ ENCCs in comparison to culture in arundic acid alone (**Figure 8**). This suggests that extracellular S100B does not play a role at these early stages of ENS development, and that the effects on Sox10 are mediated by cell-intrinsic sources of S100B.

## Discussion

**FIGURE 9:**
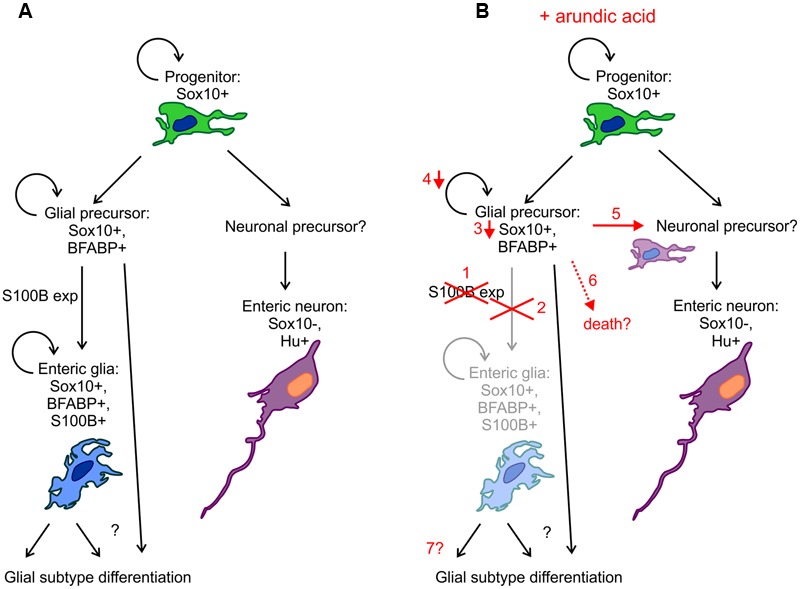
**Diagrammatic representation of the effects of arundic acid on ENS development. (A)** During “normal” development, Sox10+ ENCC progenitor cells divide and differentiation to produce neuronal and glial precursors (although the identity of the neuronal precursors remains to be examined). Glial precursors and enteric glia remain proliferative, whilst there is limited proliferation of enteric neuronal precursors and neurons. S100B expression marks the differentiation of glial precursors into glia, but how glial subtype differentiation occurs remains unknown. **(B)** Addition of arundic acid results in: (1) inhibition of S100B synthesis; (2) failure of glial precursors to differentiate into S100B+ enteric glia and a severe reduction in the population of S100B+ enteric glia; (3) glial precursors that were committed to turn on S100B expression exhibit decreases in their Sox10 protein levels; (4) and subsequently decrease in proliferation; (5) possible differentiation of some glial precursors into neurons (or neuronal precursors) thereby producing Sox10+/Hu+ ENCCs; (6) cell death is also possible; (7) the longer term changes on enteric glial differentiation remain to be examined further.

Enteric glia are an integral part of the ENS and play many roles in regulating neuronal function, as well as interacting with other cell types in the gastrointestinal tract (Bush et al., 1998; Cornet et al., 2001; Gomes et al., 2009; Gulbransen and Sharkey, 2009; Kabouridis et al., 2015). S100B, Sox10 and GFAP are common markers used to label glial cells in the mature ENS and it has been found that the combination of expression of these markers varies in different subtypes of enteric glia (Boesmans et al., 2015b). Recently, proteolipid protein 1 (PLP1) has also been shown to be expressed by enteric glia (Rao et al., 2015). In this study, we show that S100B is important for enteric glial differentiation. Using arundic acid to inhibit S100B synthesis, we found that there were (i) decreased Sox10+ ENCC density and Sox10 immunoreactivity; (ii) reduced ENCC proliferation; (iii) co-localization of Sox10 and Hu in a small subpopulation of cells; but (iv) no effect on the overall number of Hu+ enteric neurons. A diagram of our proposed model is shown in **Figure 9**. It is our hypothesis that by inhibiting S100B expression, glial precursors fail to differentiate into enteric glia and a subpopulation begin to differentiate into neurons (**Figure 9**). Our results suggest that S100B could be important for maintaining *Sox10* expression during enteric gliogenesis.

### Enteric Glia Differentiation

Glial differentiation in the ENS appears to lag behind enteric neuron differentiation, as the first appearance of the “mature” glial markers S100B and GFAP occur several days after the first detection of pan-neuronal markers at E10.5 (Rothman et al., 1986; Baetge and Gershon, 1989; Young et al., 2003). We found that the proportion of S100B+ ENCCs increases gradually during development, and similar to many other events in gut development, the differentiation of glia also appears earlier in the rostral small intestine compared to the colon. There are four distinct subtypes of enteric glia in the mature gut (Hanani and Reichenbach, 1994; Boesmans et al., 2015b), however, the differentiation of these subtypes could not be determined in our experiments as we were unable to examine individual cell morphologies. However, at E16.5, S100B+ cells that were not immunoreactive for Sox10 could mark the first appearance of glia that express subtype-specific combinations of these markers. Although GFAP and BFABP expression have been detected in the embryonic gut in previous studies (Rothman et al., 1986; Young et al., 2003), we were unable to label distinct cell bodies for individual cell counting in this study.

### Abolishing S100B in the ENS Using Arundic Acid Decreases Sox10 Levels and Reduces ENCC Proliferation

In the CNS, arundic acid has been shown to inhibit synthesis of S100B reducing the levels of its mRNA and protein in astrocytes (Tateishi et al., 2002; Asano et al., 2005). In our study, in order to abolish S100B synthesis in developing glia, we added arundic acid to embryonic gut from E13.5, the day before the first immunohistochemical detection of S100B, in *in vitro* organ culture. This culture system appears to have successfully inhibited the onset of S100B production, as almost no, or very few, S100B-immunoreactive cells could be detected in arundic acid, whilst many S100B+ cells were present in control cultures.

Culture in arundic acid resulted in decreases in the density of Sox10+ ENCCs, as well as reduced immunohistochemical levels of Sox10 within cells. We also observed decreases in the overall density of ENCCs and a decreased population of proliferating ENCCs. It is well-known that Sox10 is important for maintaining a “progenitor pool” in the ENS (Paratore et al., 2001; Kim et al., 2003). Therefore, the reduction in ENCC density and proliferation in our arundic acid cultures could be due to the decrease in Sox10. Although some ENCCs expressing pan-neuronal markers continue to proliferate during ENS development (Young et al., 2005), because arundic acid did not appear to influence enteric neuron numbers, it is unlikely that their proliferation was affected.

Interestingly, the reduction in Sox10+ ENCCs and ENCC proliferation was observed in both the duodenum and ileum of arundic acid cultures, despite limited S100B-immunoreactive glia in the ileum of control cultures. It is possible that low levels of S100B protein are present in the ileum, but are below immunohistochemical detection levels. It is also possible that arundic acid acts via an S100B-independent mechanism, as in addition to inhibition of S100B synthesis, arundic acid has also been shown to influence the levels of several other proteins (Asano et al., 2005). However, with the technical difficulties posed by transfecting and transducing the ENS in intact gut preparations (Boesmans et al., 2015a), we have not been able to use a more specific, genetic approach for inhibiting S100B expression and this pharmacological approach is currently our best method for reducing S100B synthesis *in situ*. In addition, the results from our adult ENS cultures show that with the presence of native S100B, arundic acid did not affect Sox10 levels. Therefore, while we cannot definitely show that S100B influences *Sox10* expression or protein levels, our results suggest that the effects of arundic acid on Sox10 immunoreactivity were dependent on S100B.

How the inhibition of S100B synthesis might reduce Sox10 levels is not known. S100B has many intracellular binding partners (reviewed in Santamaria-Kisiel et al., 2006) and has been shown to bind to the basic helix-loop-helix (bHLH) transcription factor E12, also known as Tcf3, to inhibit its DNA binding ability (Onions et al., 1997). Whether other similar transcription factors might be affected remains to be investigated. Another possible pathway is the binding of S100B to the tumor suppressor protein, p53 (Baudier et al., 1992). This interaction reduces p53 activity and, therefore, increases cell proliferation (Lin et al., 2004, 2010). The role of p53 in cancer progression has been extensively studied, but whether changes in p53 activity might be linked with *Sox10* expression during ENS development is unknown. Recently, it has been shown that mice with neural crest specific mutations in *Geminin*, which encodes a cell cycle dependent protein, exhibit intestinal aganglionosis resulting from increased p53-dependent apoptosis (Konstantinidou et al., 2016).

Although we saw very little cell death in our embryonic gut cultures by TUNEL staining, we cannot exclude that death of cells committed to glial differentiation contributes to the decrease in Sox10+ and GFP+ numbers (**Figure 9**). Previous studies have shown that ENCC cell death in the gut is difficult to identify using conventional assays (Uesaka et al., 2007; Enomoto, 2009; Chalazonitis et al., 2012). Therefore, while the absence of TUNEL+ cells may help confirm the lack of cytotoxicity of arundic acid, it does not exclude the possibility of ENCC cell death.

### Addition of Exogenous Extracellular S100B Does Not Rescue Sox10 Expression

Apart from its intracellular roles, extracellular S100B can also influence many aspects of glial function (Donato et al., 2009). Similar to the CNS, S100B in the gut has been postulated to provide neurotrophic support at “lower” (nanomolar) concentrations, whilst “higher” (micro-molar) concentrations of S100B appear to lead to neuronal damage, such as during gut inflammation (Cirillo et al., 2011). In our study, the addition of exogenous extracellular S100B, at either concentration, had little effect on ENCCs, and did not rescue the change in Sox10+ cell numbers. Therefore, whilst released extracellular S100B can exert influences on gene transcription in a paracrine (or autocrine) fashion, we hypothesize that the effects of S100B on Sox10 observed in our study were due to its cell-intrinsic intracellular actions. The extracellular actions and appearance of receptors for S100B could arise later in ENS development. It is also possible that the S100B protein failed to penetrate the external serosal cell layers in our embryonic gut explants, thereby, failing to contact ENCCs. However, without a clear assay to examine the effect of exogenous S100B application during embryonic development, it is currently difficult to determine whether this lack of effect was due to lack of penetration or because there are limited responses to exogenous S100B.

### Inhibition of S100B Synthesis Did Not Affect Neuronal Differentiation

We did not observe any changes in the overall density of Hu+ enteric neurons in response to S100B cell loss. However, because our embryonic gut cultures were only maintained for 48 h, we can only conclude that the initiation of neuronal differentiation was not influenced by the lack of S100B, which is in accordance with the observation that neuronal differentiation generally precedes glial differentiation. Interestingly, however, we did observe a novel population of ENCCs that express both the neuronal marker Hu and Sox10. Sox10 co-localization with neuronal markers is rarely observed in the developing ENS (Young et al., 2002; Anderson et al., 2006). A small population of Sox10+ cells near the wavefront of ENCC migration that exhibit cytoplasmic Hu immunoreactivity and weak PGP9.5 immunoreactivity are thought to be progenitors that are beginning to acquire neuronal phenotype (Young et al., 2004). In the adult ENS, cells that exhibit both neuronal and glial markers can be identified, but very rarely, and make up a very small proportion of the total population of the ENS in the mouse (Musser et al., 2015). We hypothesize that after culture in arundic acid, the Sox10 and Hu co-expressing cells are glial progenitors that, due to the lack of S100B expression and subsequent downregulation of *Sox10*, are beginning to express pan-neuronal markers (**Figure 9**). It is also possible that they are enteric neurons that up-regulate *Sox10*; however, this would suggest a paracrine action of S100B, which our results demonstrate to be unlikely at this stage of ENS development. Future lineage tracing experiments are required to determine the identity of these cells.

The effects of longer term reduction of S100B synthesis remain to be investigated further. Continued inhibition of S100B synthesis would eventually prevent paracrine (and autocrine) S100B signaling and therefore, could influence later stages of ENS development. However, in two independent lines of S100B knockout mice, there appears to be little drastic effects on the health of the animals (Xiong et al., 2000; Nishiyama et al., 2002). Although changes were observed in various aspects of their nervous system, the development of the cerebellum has been examined in detail, and did not differ from wild type littermates (Bluhm et al., 2015). However, the gut and ENS have not been examined in these mice. It is possible that functional redundancy or compensating mechanisms make up for the lack of S100B during development, which could dilute the acute effects observed in our cultures over time. Also, as S100B is not expressed by all enteric glial populations (Boesmans et al., 2015b), its effects on the development of different subtypes of enteric glia remain to be investigated further.

## Conclusion

In this study, we have been able to inhibit synthesis of S100B using arundic acid in our embryonic gut culture system. Preventing S100B synthesis led to a reduction in the number of Sox10+ ENCCs, and decreases in the overall ENCC density in the gut as well as reduced proliferation of ENCCs. Our results suggest that S100B alters levels of Sox10 protein in early differentiating glia, which is vital for maintaining their proliferative potential. The long-term effects of this depletion on later glial differentiation, enteric neuron density and function require further investigation.

## Author Contributions

EC and MH performed experiments and analysis. Study design by MH, EC, WB, and PVB. Manuscript written by MH. Manuscript revision by EC, CC, WB, and PVB.

## Conflict of Interest Statement

The authors declare that the research was conducted in the absence of any commercial or financial relationships that could be construed as a potential conflict of interest.
